# Selenium as an important factor in various disease states - a review

**DOI:** 10.17179/excli2022-5137

**Published:** 2022-07-05

**Authors:** Marek Kieliszek, Iqra Bano

**Affiliations:** 1 Department of Food Biotechnology and Microbiology, Institute of Food Sciences, Warsaw University of Life Sciences, Nowoursynowska 159 C, 02-776 Warsaw, Poland; 2 Department of Veterinary Physiology & Biochemistry, Shaheed Benazir Bhutto University of Veterinary and Animals Sciences Sakrand (SBBUVAS), 67210, Sindh, Pakistan

**Keywords:** selenium, antioxidant, oxidative stress, selenoproteins

## Abstract

Selenium (Se) is an element that has a pro-health effect on humans and animals. However, both the deficiency of this element and its excess may prove harmful to the body depending on the chemical form of the selenium, the duration of supplementation, and the human health condition. Many data indicate insufficient coverage of the demand for selenium in humans and animals due to its low content in soils and food products. A balance in the physiological process of the body can be achieved via the proper percentage of organically active minerals in the feed of animals as well as human beings. Selenium is a trace mineral of great importance to the body, required for the maintenance of a variety of its processes; primarily, selenium maintains immune endocrine, metabolic, and cellular homeostasis. Recently, this element has been emerging as a most promising treatment option for various disorders. Therefore, research based on Se has been increasing in recent times. The present review is designed to provide up-to-date information related to Se and its different forms as well as its effects on health.

## Introduction

Currently, selenium is one of the most important and intensively studied micronutrients. This element was discovered in 1817 by the Swedish chemist J.J. Berzelius, in the course of research on a new method of producing sulfuric acid. During sulfur combustion, a red-brown sludge obtained from pyrite (iron sulfide) from a mine in Falun, Sweden had been observed. Initially, this distinctive precipitate was considered to be the most toxic compound - arsenic, therefore, processing of pyrite from Falun was avoided. However, the phenomenon was found to be interesting and was re-analyzed. During subsequent studies, it was found that the sediment contained a new, previously unknown compound with properties similar to tellurium. Referring to the similar properties of tellurium, whose Greek name means Earth (Tellus), selenium was given the name meaning the Moon (Tsuji et al., 2021[67]). Scientists became interested in selenium when it was discovered in the 1950s that increased selenium accumulation causes muscular dystrophy, and a deficiency of this element and vitamin E causes acute liver necrosis in studied rats (Duntas and Benvenga, 2015[15]). In 1973, selenium was discovered to be an important component of the active center of the enzyme glutathione peroxidase. After less than 20 years, researchers found that other enzymes also contain a selenium atom in their active centers. For example, selenocysteine builds the active center of iodothyronine deiodase. These discoveries and the recognition of many selenoproteins and selenoenzymes were the impetus for researchers to start intensive research on the importance of this element for the human body (Kieliszek, 2019[28]). The eighties and nineties of the twentieth century were spent on research into determining the total content of selenium and its other forms in biological materials. The results of these studies were the starting point for explaining the metabolism of selenium compounds and calculating the daily selenium requirement for the human body. Trace mineral supplementation is crucial for the maintenance of animal and human health. Several trace minerals serve as enzymatic cofactors and metallic enzymes in various biological systems (Vural et al., 2020[69]). As a general rule, they activate enzymes that participate in the removal of cellular free radicals from the body. The endocrine system, as well as metabolism, is directly influenced by several of these minerals, which are also key components of some hormones. Thus, any change in their concentration could influence the synthesis of other hormones involved in the maintenance of reproductive systems (Mirnamniha et al., 2019[41]; Arshad et al., 2021[2]; Barchielli et al., 2022[6]). Selenium (Se) is one of the major trace minerals, placed in the 34^th^ position in the periodic table. A growing number of researchers are focusing on the role of Se in the preservation of a wide range of bodily processes, which has led to an increase in interest in Se research. Some experts believe that this component plays a key role in the longevity of male fertility, serves as a regeneration agent, and has consequences for the endocrine system of the animal body via maintenance of ratios of various antioxidant factors such as several enzymes and by-products including glutathione peroxidase (GPx), superoxide dismutase (SOD), malondialdehyde (MDA), and catalase (CAT) (Barchielli et al., 2022[6]; Kieliszek et al., 2022[29]). According to previous studies, it has been proven that Se also has major effects on somatic growth in mammals and birds by influencing the insulin growth-like factor axis (IGF), maintenance of triiodothyronine (T3), tetraiodothyronine (T4), thioredoxin reductase (TrxR), and growth hormone (GH), respectively. Research into the regulation and functional characterization of selenoproteins (SelPs) has helped researchers better understand how Se affects human health as well as the wide range of physiological processes that are affected by this trace element (Kieliszek and Błazejak, 2013[30]). The major SelPs and their functions in the body are elaborated in Table 1(Tab. 1) (References in Table 1: Bertz et al., 2018[9]; Gomes Alves Andrade et al., 2021[21]; Mangiapane et al., 2014[38]; Negro, 2008[48]; Ogawa-Wong et al., 2016[50]; Pothion et al., 2020[53]; Saito, 2020[62]; Verma et al., 2011[68]; Yang and Liu, 2017[76]; Zhang et al., 2021[85]). Eukaryotic nuclear SelPs protect the genome from OS (oxidative stress) by scavenging free radicals. There are now more than 50 families of SelPs recognized, most of which were discovered using bioinformatics techniques. SELENOP seems to be the only SelP believed to be confined to the nucleus among the others (Ha et al., 2019[22]). Some SelPs belonging to the GPx and TrxR families that are especially vulnerable to a probable dietary Se deficit, which may be related to a lower expression of some SelPs. Collectively, the SelPs described above control redox stability and protein quality. According to recent research, the availability of Se varies greatly throughout the European countries with some states lacking and others oversupplied (Benhar, 2018[8]). The health benefits and illnesses connected to a shortage of Se are explored in the current review.

## Different Forms of Selenium

**Figure 1:**
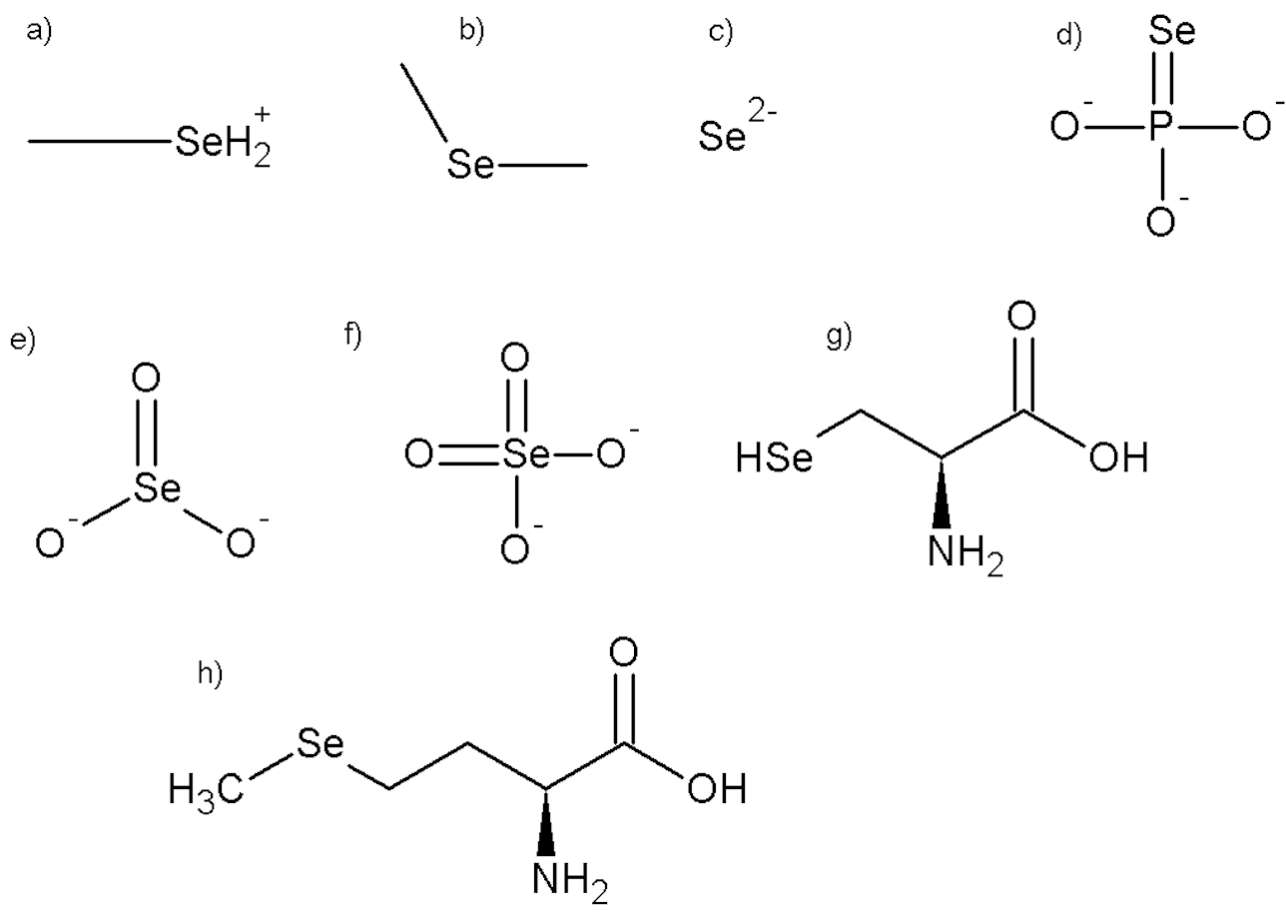
The chemical structures of different selenium compounds (a: monomethylselenonium, b: dimethylselenide, c: selenide, d: seleniumphosphate, e: selenite, f: selenate, g: selenocysteine, h: selenomethionine)

Selenium is a micronutrient, and like other elements, circulates in nature, and Se can be found in two distinct forms; inorganic and organic. The in-organic forms include selenate (Na_2_SeO_4_) and selenite (Na_2_SeO_3_) (Kieliszek and Błazejak, 2013[30]), whereas the organic form includes selenomethionine (SeMet) and selenocysteine (SeCys). Both forms of Se are known to be effective dietary sources of the mineral. In-organic Na_2_SeO_3_ and Na_2_SeO_4_ are found in soils and are accumulated by plants, which convert them to organic forms as well as their methylated derivatives. It is estimated that skeletal muscle stores between 28 to 46 % of the total Se pool, making it the most important site of storage (Hariharan and Dharmaraj, 2020[23]). It is possible to decrease SeCys and Na_2_SeO_3_ to produce hydrogen selenide, which is then transformed to selenophosphate for use in SelPs biosynthesis. On the other hand, Se is found in higher animals and humans in the form of SeMet, which replaces the methionine in plant proteins (Hu et al., 2018[24]). Rather than methionine, the body uses SeMet which is more easily absorbed and may be metabolized or incorporated into protein (Gandin et al., 2018[20]). As methionine intake increases, SeMet incorporation is diminished. It is mostly present in the skeletal muscle, erythrocyte, pancreas, liver, stomach, kidney, and gastrointestinal mucosa proteins; its release from body proteins is associated with protein turnover and occurs continuously. When SeMet intake is kept constant, a steady state is formed and may be maintained throughout a broad range of intakes for all times (Roman et al., 2014[60]). The chemical structures of different forms of Se are elaborated in Figure 1(Fig. 1).

## Sources of Selenium

Selenium is found in the atmosphere, hydrosphere, lithosphere, and biosphere of the earth. Depending on the type of environment, selenium is present in different concentrations. Naturally occurring selenium comes from the weathering of volcanic rocks and the emission of dust into the atmosphere. Additionally, by decomposing organic matter rich in selenium, microorganisms enrich the atmosphere with selenium compounds (Mehdi et al., 2013[40]).

Regardless of soil thickness, the Se concentration in mineral soil is approximately 14 mg/kg. Only trace amounts of Se are found in groundwater, while the concentration of Se in seawater can rise dramatically. Se extraction from source rocks and run-off by intensive soil fertilization with combinations heavy in Se compounds are the primary causes of seawater's greater content of Se (Bano et al., 2021[5]). There is a limit to how much Se can be safely ingested by humans according to World Health Organization (WHO) recommendations. Several factors influence the amount of Se in food such as the soil and cultivating situations in which bread and cereal crops are grown, the forage that animals eat, and the refining of these commodities for human consumption, all of which affect the amount of Se present in the final product. Also, Se may be found in both organic and inorganic chemical forms in foods and biological materials. When it comes to bioavailability, the chemical form of Se may have an impact on how it is absorbed; SeMet is more bioavailable than inorganic Na_2_SeO_4_ or Na_2_SeO_3_ because it is organic (Rosetta and Knight, 1995[61]). The efficiency of selenium absorption is dependent on the form in which SeMet> MeSeCys>Se (VI)>Se (IV) occurs (Thiry et al. 2012[64]). Many nations have set dietary standards to guarantee appropriate Se consumption for the sake of human health (Tinggi, 2008[65]). Based on the research by (Huang et al., 2013[25]) on the relationship between the doses of this element and the occurrence of clinical selenosis and its symptoms, EFSA experts established the UL level (upper tolerable level of consumption) for this element and set the UL value at 300 μg/day for adults. The maximum tolerable level of daily consumption includes the provision of selenium with both food and supplements. For children and adolescents, this value was calculated on the basis of body weight and reduced accordingly. Both the consumption of selenium in excess and its deficiency are toxic to the human body and may have adverse health effects (Rayman, 2017[56]). If the recommended dose for consumption by an adult is exceeded for a long time by more than 300 μg/day, a disease called selenosis may develop (Petrović, 2021[52]). In this case, the body reacts by producing weakening and brittle nails. It is also associated with substantially increased hair loss. There may be general weakness and fatigue of the body, mental disorders, e.g., depression or nervousness. Excessive consumption may also be accompanied by gastrointestinal disorders and skin lesions. If the dose of selenium is well above the standard, there is a risk of disturbing the functioning of internal organs at one time, which may lead to cirrhosis of the liver or even pulmonary edema (MacFarquhar et al., 2010[37]).

Selenium in water, soil, and air is accumulated in plant tissues and thereby introduced into the food chain. Inorganic selenium found in plants is less digestible than organic selenium from animal tissues, and products of animal origin are, therefore, a better source of selenium, i.e., meat, fish, and dairy products. Animal products are considered the basic source of this micronutrient in the diet of the European population. People who do not eat meat satisfy their selenium needs through nuts - mainly Brazil nuts and mushrooms (Chen et al., 2021[12]). Cereal products and some vegetables and fruits are also high in this micronutrient. The most susceptible to selenium accumulation are cruciferous vegetables (white cabbage, Brussels sprouts, cauliflower) and garlic vegetables (garlic). However, it should be remembered that the content of selenium in plant products is related to the amount of this element in the soil in which the plant is grown. The soils of all of Europe, including Poland, are characterized by a low content of this element (Mirończuk-Chodakowska et al., 2019[42]). This means that plant-based products are not the main source of selenium in our diet. It is also worth noting that in the case of soils fertilized with selenium compounds, the selenium content in plant tissues will be higher (Izydorczyk et al., 2021[27]).

There are huge differences in soil Se levels across the globe, and these large variations in soil Se levels are mirrored in the wide variances in the Se status of human populations (Yamashita et al., 2013[74]). Several nations have now successfully implemented a breakthrough technological procedure aimed at processing Se-rich food products such as eggs, beef, and dairy. The Korean market has pork and chicken boosted with Se, while eggs fortified with Se are currently available in 25 nations across the world. To fill any micronutrient deficiency and maintain the body's metabolic equilibrium, it is clear that eggs enhanced with Se might be employed as functional meals (Bano et al., 2021[5]).

## Selenium Supplementation

Recommended dietary intakes of Se and other minerals are described in the dietary reference intakes (DRIs) established by the Food and Nutrition Board (FNB) at the Institute of Medicine of National Academies. The term "DRI" refers to a collection of reference values that are used to plan and assess the nutritional intakes of healthy persons regularly. These values differ depending on one's age and gender (Yates, 1998[80]). In different parts of the world, DRI and tolerated upper intake levels (UL) for Se differ. For example, in the United Kingdom (UK), men should consume 75 g/day and women should consume 60 g/day. Moreover, the European Food Safety Authority (EFSA) recommends a 55 g/day Se intake. The overconsumption of Se can cause selenosis, a hazardous condition in which the body becomes overexposed to Se (Stoffaneller and Morse, 2015[63]). It is worth noting that studies have already been conducted on the health effects of taking excessive amounts of this element in the form of a dietary supplement with a dose of 41.749 µg/day. 227 people participated in the study, and their symptoms varied greatly; only 58 % of the respondents felt nauseous, and a little more - 61 %, noticed discoloration of their nails and increased brittleness. A majority of people participating in the study complained about diarrhea (78 %), chronic fatigue (75 %), and increased hair loss (72 %), while pain in the joints affected as many as 70 % of respondents. The vast majority of symptoms disappeared after the end of supplementation. However, symptoms such as fatigue and hair and nail brittleness persisted up to 90 days after the end of the study (MacFarquhar et al., 2010[37]). The Se might also become pro-oxidant at even higher quantities, resulting in oxidative stress (OS) and cell damage. As a result, it is critical to keep the body's Se concentration at a healthy level while also avoiding the harmful consequences of an overabundance of the mineral (Xia et al., 2021[73]). In the early 1970s, regulatory bodies needed to evaluate which Se compounds may be used in animal feed, but nothing was known about SeMet. Na_2_SeO_4_ and Na_2_SeO_3_ were approved as feed additives in 1974, however, the situation was unsatisfactory so, just because Se-rich foods have been authorized by the Food and Drug Administration (FDA) does not always guarantee they are healthy options. When these permissions were granted, Se compounds such as SeMet were also missed (Yang et al., 2022[77]), and the only Se compounds available for animal feed at the stage of the regulatory action were the inorganic Se compounds. The first commercially accessible "high SeYeast" appeared in the mid-1970s. With 90 % of the Se found in commercial goods being in the form of SeMet, these products generally included between 1,000 and 2,000 μg of Se/mg (Tsuji et al., 2021[67]). Large-scale cancer prevention trials began in 1983 using this SeMet-Yeast as the Se source. An additional 200 mg of Se/day dramatically reduced the chance of getting prostate, lung, and colorectal cancer in this study. The FDA authorized the use of SeYeast in chicken broiler and layer feeds in June of 2000 and a lengthy process of research and development will lead to SeMet or other nutritional sources ultimately replacing inorganic Se compounds as feed additives (Lyons et al., 2007[36]). Since Brazil nuts are recognized to be one of the largest sources of SeMet, they have been employed extensively in the study of Se supplementation. Regular intake of Brazil nuts leads to optimal plasma Se and erythrocyte concentrations as well as improved efficiency of selenoenzymes antioxidant state, muscle retention, and inflammatory status (Roman et al., 2014[60]). Before beginning clinical trials, it is critical to take into account genetic variations in SelPs genes as well as to pre-stratify the population to prevent potentially varied reactions based on the Se status of each person. The nutritional Prevention of Cancer (NPC) experiment showed that SeYeast (200 mg/day) may reduce the incidence of malignancies of the uterus, prostate, lung, and colon. Moreover, the Se supplementation in the form of SeYeast (200 g/day) dramatically boosted Se levels in healthy New Zealand males and improved DNA stability (Ferreira et al., 2021[18]).

In cases of using selenium yeast as a feed additive and dietary supplement, not even a single accidental poisoning with this element was reported, and lower chronic toxicity compared to sodium selenate was found. The first selenium yeast production process was developed over 30 years ago and, initially, the quantification of selenomethionine was difficult due to the poor characteristics of yeasts and their composition. Over time, with the use of improved methods of analysis, the composition of the yeast has been found to be more uniform than initially assumed. It is selenomethionine that is the main form of selenium in yeast cells, therefore, they can be treated as an excellent source of naturally synthesized food form of selenium (Loef et al., 2011[33]).

In 2012, the European Food Safety Authority (EFSA) Panel on additives and products or substances used in animal feed issued a positive opinion on the safety and efficacy of selenium in the selenium yeast *Saccharomyces cerevisiae* NCYC R646 (Selemax 1000/2000) as a feed additive for animals of all species. The Panel on Additives and Products or Substances used in Animal Feed (FEEDAP) states that supplementation should not exceed 0.2 mg of selenium per kg of complete feed. Such dosing will ensure the safety of consumers against consuming tissues and products of animal origin that have consumed the preparation. Additionally, Selemax is believed to be an effective source of selenium and does not change the quality of meat measured by physical parameters. Due to the lack of data, the product is considered to be potentially irritating to the skin and eyes as well as being skin sensitizing, and due to its proteinaceous nature, it is considered a potential respiratory sensitizer (EFSA, 2012[16]).

In turn, in 2020, the Panel of the European Food Safety Authority (EFSA) for additives and products or substances used in animal feed renewed the permit for the use of selenium-enriched yeast produced by *Saccharomyces cerevisiae* CNCM I-3399 as a feed additive for all animal species. It was again found that the use of the additive in the permitted amounts is safe for target species and consumers as well as the environment (Bampidis et al., 2020[3]). 

## Selenium Status in Various European Countries

In Europe, Se intakes tend to be lower than in the US, due to soils being a less reliable supply of the mineral. To assess whether these levels are enough or not, we must first establish acceptable benchmarks against which to measure them, however, this subject has divided opinion. There has been a steady fall in the UK's Se intake since the 1970s, and prior government studies show that Se consumption is low across the UK population as a whole (Rayman, 2002[57]). According to a Polish study, the Se level of the foods consumed in Eastern Europe was four times lower than the Se content in Spain, which appeared to surpass the DRI and recommended daily allowance (RDA) levels of the nutrient. Research conducted in France and Belgium found intakes comparable to the RDA, while studies conducted in Slovenia and Italy found intakes lower than the RDA. In Europe, the results of Se status investigations show that most populations have blood Se concentrations that fall short of the required amount for complete plasma GPX expression (Stoffaneller and Morse, 2015[63]), with a few notable exceptions, including Austria, Hungary, Denmark, Poland, and some of the participants in the "IMMIDIET" study, which examined the impact of migration on dietary habits in European communities to the varying risk of coronary heart disease as a model of gene-environment interaction (Iacoviello et al., 2001[26]). The reported serum Se levels in Albanian individuals residing in Greece had the highest concentration of Se in all the European studies, at 37.4 g/L, wherein, insufficient animal protein intake could be the cause. The Se concentration in Estonian soils was studied and results revealed a mean value of 0.172 mg/kg (ranging from 0.010-0.443 mg/kg) (Stoffaneller and Morse, 2015[63]). Moreover, another research suggested that Se insufficiency and the plant-animal food chain, are linked. For example, blood and milk samples taken from Estonian dairy cows were discovered to be deficient in Se (Rauhamaa et al., 2008[55]). Serum or plasma Se levels are closely linked to erythrocyte GPx activity when Se consumption is low or moderately low. In populations with low or moderate Se consumption, serum or plasma Se serves as a helpful measure of status, notwithstanding the limitations outlined above. Europe has a similar scenario; Se levels are too low for GPx activity to be fully saturated (Nève, 1995[49]; Demircan et al., 2021[13]). There is some evidence to suggest that consuming enough Se to maximize immune response and minimize cancer risk is not enough to reach levels that meet the enzymic or antioxidant function of Se in plasma. Se intake of less than two-thirds of the recommended daily value would exacerbate this deficiency even more. Functional Se markers, which indicate physiologically effective concentrations, are being explored. The potential of Se from SeYeast to be preserved in the organism and reversibly removed by various metabolic processes to counteract periods of insufficient intake is likely to be particularly valuable in areas of low Se intake such as those found in Eastern Europe. Se from SeYeast can be stored in the organism and transiently cleared by normal metabolic processes. The sale of SeYeast in Europe should not be prohibited as a result, especially if the criteria for upper safe limits (such as the somewhat conservative European Community tolerated maximum intake threshold of 300 mg/d) are followed (Rayman, 2004[58]).

## The Role of Selenium in Different Diseases

**Figure 2:**
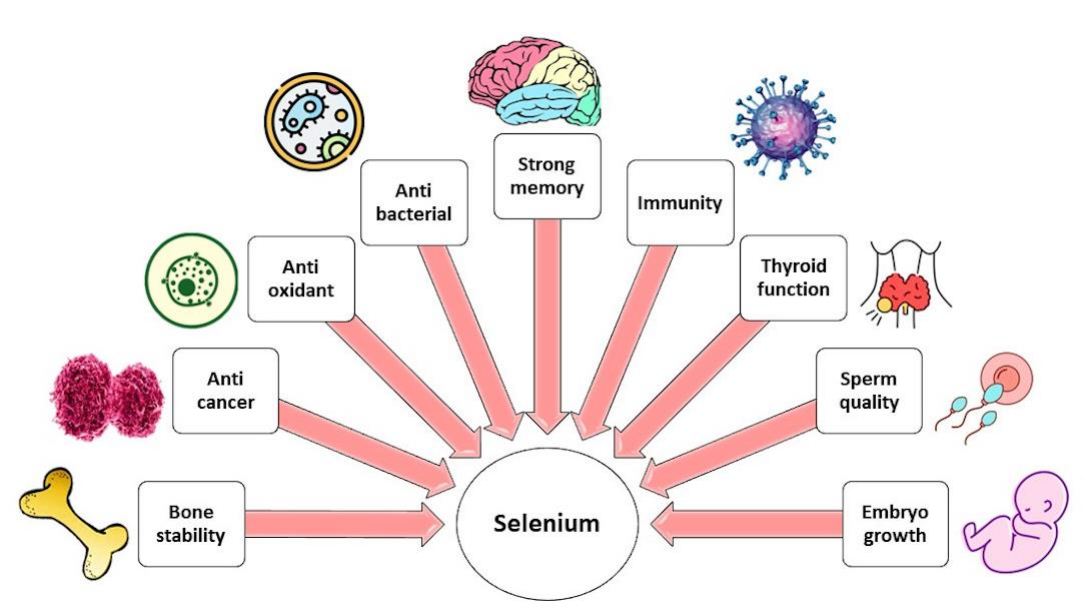
Effects of selenium on various health conditions

In normal functioning, Se plays an important role and participates in the pathogenesis of a wide range of illnesses (Figure 2(Fig. 2)). A healthy diet rich in Se appears to protect against a wide range of diseases, including cancer, cardiovascular disease, neurodegenerative disease, and problems with fertility, by maintaining the body's Se-dependent redox homeostasis. This is accomplished, in part, through the production of antioxidant SelPs. On the other hand, even though excessive Se intake may lead to toxicity, mental problems, and cancer, supra-nutritional dosages of Se compounds can be used as chemotherapeutic agents for their pro-oxidant and pro-apoptotic effects on cancer cells (Barchielli et al., 2022[6]).

### Selenium as an antioxidant

Oxidative stress (OS) is a state that occurs when a system's capacity to neutralize and remove reactive oxygen species (ROS) is outstripped. ROS are byproducts of cellular metabolism, principally created by electron leakage from mitochondrial electron acceptors and enzymes throughout oxidative phosphorylation (Marín et al., 2020[39]; Wang et al., 2021[71]). Overproduction or distribution of ROS from endogenous sources or external stress may lead to an antioxidant capacity deficiency, which in turn can generate an imbalance. Damage to lipids, proteins, or DNA might impede signal transduction pathways and overall cellular function if ROS levels are not appropriately managed (Roman et al., 2014[60]). As a result, OS has been linked to a wide range of human disorders, including cardiovascular and neurological diseases, cancer, and the aging process. Chemical compounds that prevent ROS from forming or reacting with biological structures are known as antioxidants. Enzymatic catalysis may be used to convert inorganic Se molecules like Na_2_SeO_3_ and Na_2_SeO_4 _to organic forms and vice versa. ROS signaling has two main modes of action, namely, changes in intracellular redox status and protein oxidative modifications (Tsuji et al., 2021[67]). OS may be reduced by GPx and TrxR, which work as thiol-redox systems to reduce H_2_O_2_ and lipid hydroperoxides in the body. One of the most important aspects of Se is its involvement as a component of numerous critical antioxidant compounds as well as the particular oxidation properties of the antioxidant molecule thioredoxin reductase. GPX reduces ROS metabolites to protect membrane integrity (Tinggi, 2008[65]). Research into the effects of Se and SelPs will aid in the development of novel medicinal approaches; more specifically, ebselen, an organo-Se compound that mimics glutathione peroxidase, has been shown to suppress superoxide anion formation and release of NO as well as to scavenge peroxynitrite and protect against lipid peroxidation, which is consistent with its proposed ability to prevent the onset of OS (Zarczyńska et al., 2013[83]).

### Selenium for brain disorders

Downregulation or damage to Se and SelPs, which play a crucial physiological role in neurons, astrocytes, and microglia, may result in brain dysfunction. The Se levels in the brain decline as we age, and this decline is linked to cognitive decline (Whanger, 2016[72]). Moreover, Se has a role in the prevention and treatment of Alzheimer's disease (AD), either alone or in conjunction with other factors. When comparing AD patients to healthy controls, one study found a clear link between lower Se plasma concentrations and cognitive impairment. In the early stages of AD, the reduction in plasma Se levels was not related to the dietary condition**.** Another research suggested that the AD brain tissue's Se levels were also markedly lowered, particularly in the hippocampus and in the frontal, parietal, temporal, and occipital lobes (Loef et al., 2011[33]). In addition, it was shown that Se therapy had a positive impact when combined with other neuroprotective substances (Barchielli et al., 2022[6]). Na_2_SeO_3_ and natural carotenoid dicarboxylic acid, when used together, offered superior neuroprotection in treated rats with streptozotocin (STZ) by lowering lipid peroxidation and increasing GSH, GPX, glutathione S-transferase (GST), and CAT activity (Dominiak et al., 2016[14]). Several *in vitro* experiments have shown that Se protects the brain against poisons that cause Parkinson's disease symptoms to persist indefinitely in the body. Besides, the formation of reactive nitrogen species (RNS) was also decreased, and the lowering of GPx levels in dopaminergic neurons produced by methamphetamine (MA) was ameliorated by Se supplementation (Navarro-Alarcon and Cabrera-Vique, 2008[47]). As a result of a lower serum and erythrocyte Se concentration in epileptic patients, it was previously believed that Se use may be enhanced. The depletion of Se in the brain during epilepsy is also thought to be a significant component in the onset of seizures (Dominiak et al., 2016[14]). As the OS is frequently accompanied by a loss of vital trace elements in patients with cerebral ischemia, it is also worth noting that Se levels were considerably lower in the ischemic brain compared to the control participant. Ischemia and reperfusion damage might benefit from the scavenging properties of Se, hence therapy utilizing Se-derived compounds was recommended (Whanger, 2016[72]). The prefrontal cortex and hippocampus of a rat model of ischemia/reperfusion were shown to have increased neuron density and reduced perineuronal and pericapillary edema after treatment with Na_2_SeO_3_ according to more current results based on histological examinations. Furthermore, the same study found that inorganic Se treatment significantly decreased the levels of inflammatory cytokines such as interleukin-1 beta (IL-1 beta) and tumor necrosis factor alfa (TNF-alfa) while simultaneously increasing the levels of neurotrophic factor (NGF) in the prefrontal cortex and hippocampus (Ramos et al., 2015[54]).

### Selenium and thyroid diseases

In comparison to other endocrine organs, the thyroid contains the highest Se content, suggesting the importance of the thyroid's actions. The maintenance of proper Se status in humans is essential for the preservation of thyroid health, the metabolism of thyroid hormones (TH), and the prevention of thyroid disorders. Numerous clinical studies have demonstrated that Se supplementation has anti-inflammatory benefits for patients with autoimmune thyroiditis, which is characterized by decreased anti-thyroid peroxidase supplement autoantibody (TPOAb) levels and restoration of thyroid function (Triggiani et al., 2009[66]). The maintenance of an optimal physiological concentration of Se is, therefore, critical to guaranteeing appropriate thyroid function and, as a result, the generation of essential regulators important to metabolism. Several biological functions of Se in the thyroid are known, including accelerating enzymatic redox processes, regulating thyroid hormone metabolism, and guarding against oxidative DNA damage caused by H_2_O_2_ and lipid hydroperoxides as well as inflammation. Single nucleotide polymorphisms in SelPs genes are related to higher risk and mortality of thyroid-associated disorders, which reflects the importance of SelPs to thyroid health (Tinggi, 2008[65]). The polymorphisms of the GPX3 are one example of a polymorphism that is related to differentiated thyroid carcinoma. Moreover, Se shortage of moderate severity has been associated with impaired thyroid function as well as an increase in the prevalence of thyroid disorders. This is because a shortage of Se results in a decrease in both deiodinases (DIO) and GPX enzymatic activity. Tetraiodothyronine (T4) is converted to its activated form, triiodothyronine (T3) by the enzyme DIO, which becomes less active as a result of the decreased activity of DIO, which results in decreased active TH production. Furthermore, a low Se status is related to a greater risk of autoimmune thyroiditis, Grave's disease, and goiter (enlargement of the thyroid gland) in women. It is now well established that Se supplementation can have a clinically beneficial effect on people suffering from autoimmune thyroiditis and Grave's orbitopathy (Mojadadi et al., 2021[45]).

### Selenium for reproduction

The ability to reproduce at the highest level is dependent on several factors, including genetics, external environmental factors, and an individual's food. Micronutrients are particularly important in the diet since they are required for a variety of biological processes, including growth and reproductive capacity. Furthermore, even minor variations in micronutrient concentrations can have a significant impact on critical physiological processes such as fertility (Zarczyńska et al., 2013[83]). According to certain research, there is a link between Se level and reproductive function in both men and women. Female reproductive health is comprised of several consecutive phases that result in the generation of an optimally functioning egg. One of the most important steps is folliculogenesis, the process by which primordial ovarian follicles in birth evolve into mature ovarian follicles after puberty (Kieliszek and Błazejak, 2013[30]). The multiplication of granulosa cells is a critical phase in the formation of folliculogenesis, and Se has been demonstrated to regulate the progression of granulosa cells as well as the manufacture of one of the key female sex hormones, 17-estradiol (E2). It has been demonstrated in a small number of studies that a connection between Se status, female fertility, and Se-dependent catalytic interaction has been established (Mojadadi et al., 2021[45]). In general, these studies have found that low serum and follicular fluid levels are associated with a higher occurrence of infertility in women. It has been shown that Na_2_SeO_3_ not only promotes oocyte growth but also increases the rate of cell proliferation in theca and granulosa cells. In support of this concept, an *in vitro* investigation conducted by Basini and Tamanini (2000[7]) showed that Na_2_SeO_3_ (5 ng/mL) treatment induced the production of nitric oxide (NO). This compound stimulated the expansion of bovine granulosa cells while also having some stimulatory effects on the production of E2. These consequences could be mitigated, at least partly, by suppressing the generation of NO in the body (Friedman, 2011[19]). Se is essential for the normal production of sperm cells as well as for the maturation of spermatozoa in mammals. When Se levels are either too high or too low, sperm production always suffers. The maturation of spermatozoa is critical to the quality of semen and male fertility, hence any interruption in this process might result in lower semen quality and infertility. Testicular structures in male goats have been shown to be influenced by Se supplementation; anomalies were apparent in the mitochondrial gaps, tail, plasma membrane, and midpiece of spermatozoa from boars fed an Se deficient diet. Overall, an insufficient supply of dietary Se leads to poor quality semen, which eventually leads to infertility since SelPs in the testis is involved in spermatogenesis (Bano et al., 2019[4]).

### Selenium and embryo 

The significance of Se in maternal nutrition, as well as its impact on the Se status of offspring, has recently attracted a great deal of attention. In vertebrates, Se is delivered to the fetus and infant through the placenta, colostrum, and milk. Among bird species, Se is transferred to the egg and then passes on to the growing fetus and freshly fledged chick as well as to the mother and her eggs (Pappas et al., 2019[51]). Se affects both non-enzymatic and enzymatic antioxidant defense mechanisms, assisting in the development of a robust antioxidant defense for both the mother as well as the developing embryo. Recent human research has also demonstrated a link between parental Se status and particular outcomes in early childhood, which is consistent with previous findings. It has been shown that both higher and lower levels of cord serum Se have detrimental impacts on an infant's neurobehavioral development (Yang et al., 2013[78]). Moreover, the impact of Se on large animals has been studied extensively, however, most of that research has focused on early gestational stages, with only a few studies looking at later outcomes. In humans, studies have focused on the effects of Se on nutrition, with only a few looking at later consequences. Small intestine weight was increased in six-month-old lambs generated from ewes fed with supranutritional Se and artificially reared to minimize confusing effects with colostral Se, but this was not accompanied by high jejunal cell proliferation (Yunusova et al., 2013[82]). In pigs, parental supplementation with SeMet greatly enhances litter weight at weaning, and in chickens, the addition of Se in the diets could positively affect embryo survivability, hatchability, and development of the offspring (Kieliszek and Błazejak, 2013[30]).

### Selenium and cancer

Selenium is of great interest in the treatment and prevention of cancer (Kieliszek et al., 2017[29]). In some cases, this micronutrient shows an antagonistic relationship between selenium consumption and cancer development, such as ovarian, pancreatic, bladder, and lung cancer. However, the therapeutic use of selenium in cancer is a moot point. The mechanisms leading to the death of neoplastic cells depend on the form of selenium, the dose used, the duration of action, and the characteristics of the neoplastic cells. Due to the specificity of the discussed microelement, it is referred to as "an element with two faces". Selenium shows antioxidant properties in small doses, and pro-oxidative properties in large doses (Wallenberg et al., 2014[70]). Low selenium concentrations protect both healthy and neoplastic cells. Cells are protected against toxicity caused by oxidative stress and support DNA repair. On the other hand, a higher concentration of selenium reduces the risk of carcinogenesis and all kinds of cellular mutations. Selenium has a significant impact on the expression of genes responsible for inflammatory responses and the remodeling of the cytoskeleton (Misra et al., 2015[43]). These are processes related to the risk of cancer incidence. *In vitro*, selenium inhibits the migration of neoplastic cells and has an anti-angiogenic effect, i.e., it prevents the formation of new blood vessels, which is characteristic of malignant neoplasms. In practice, inhibition of cellular mobility means preventing the development of tumor metastasis. This relationship has been confirmed in the case of breast, prostate, colon, or lung cancer, and in the case of lymph node metastases. Although the relationship between selenium deficiency in the blood and increased cancer incidence has been repeatedly demonstrated, little is known about the anti-cancer mechanism of this element. Selenium is used in anti-cancer therapy due to its strong anti- and pro-oxidative properties. In cancer cells, the pro- and antioxidant balance is disturbed because numerous reactive oxygen species (ROS) are produced in the process of glycolysis and the pentose cycle. The way selenium acts on cancer cells involves the production of ROS and modification of the thiol group. This procedure brings about effects that disrupt transcription and changes related to the endoplasmic reticulum (Zhao et al., 2020[86]; Razaghi et al., 2021[59]). It is worth noting that selenium may be helpful in the treatment of advanced forms of cancer through its cytotoxic effect that damages cancer cells. Selenite (IV) is used to support the treatment of cancer in many organs, including the lungs, uterus, and prostate. Selenite has been shown to have the potential to potentiate its effect on developed prostate tumors (Fernandes and Gandin, 2015[17]).

Se has been studied in human clinical studies around the world at this point. In China, the first human trials to cure cancer with Se have been conducted. About 20,847 people received Na_2_SeO_3_ which provided about 30-50 mg of Se each day for eight years. Primary liver cancer cases have dropped considerably (Yuan et al., 2022[81]). Serum Se levels and the presence of breast cancer have been linked, and these authors suggest using Se concentrations as a predictor for breast cancer. Serum Se concentrations were considerably lower in breast cancer patients compared to healthy women in a case-control study (Charalabopoulos et al., 2006[11]). GPX1 enzyme activity decreased when SelPs levels were increased in colon-derived HCT116 cells and MCF-7 breast cancer cells, according to another study. When administered orally for just 24 hours, SelPs induced a significant increase in plasma and erythrocytes concentration, plasma oxygen radical absorbance capacity (ORAC) levels, and erythrocytes Se concentration, while a decrease in thioredoxin reductase 1 (TXNRD) activity and an increase in MDA level were observed following 28 days of treatment. Moreover, previous research has also suggested that the plasma and serum Se levels are typically reduced in cancer patients. In human lung cancer cells, the SeMet has been shown to activate the tumor suppressor protein p53 by transforming oxidized p53 into the reduced form of p53. This may help guard against cancer (Abdulah et al., 2005[1]). Besides this, some plant-based Se compounds have also recently been studied for anticarcinogenic properties, with researchers particularly interested in garlic, onion, and broccoli. Moreover, chemotherapeutic drugs can be used in combination with Se to protect patients against the toxicity of the treatment. Several chemotherapeutic drugs (irinotecan, fluorouracil, oxaliplatin, and cisplatin) had their maximum tolerated dosage (MTD) increased when SeMet and SeCys were added to the treatment (Yuan et al., 2022[81]). In order to maintain the cancer cell selectivity of Se absorption, higher dosages of the Se molecule may be required. There is still a lot of work to be done in determining the optimum doses for cancer treatment that are safe and effective (Barchielli et al., 2022[6]). According to studies conducted by Kuria et al. (2020[32]), selenium in the recommended daily dose of at least 55 μg reduces the risk of cancer in adults. The Recommended Dietary Allowances vary according to age, for pregnant women, and while breastfeeding. For the proper course of physiological processes, this element is necessary for the body in small amounts. Breastfeeding women are advised to consume 70 μg of selenium per day, while children aged 1 to 3 years old require a lower amount of selenium, 20 μg. Children over 14 years of age and adults require 55 μg of selenium per day (Kuria et al., 2020[32]).

### Selenium and immunity

Immune system cells such as macrophages, natural killer (NK) cells, neutrophils, and T lymphocytes rely on Se to do their jobs properly. OS, inflammation, and the spread of infectious diseases can all be alleviated or even prevented with a suitable rise in serum Se concentration in the diet (Roman et al., 2014[60]). Immunoglobulin production is increased by Se, which promotes the differentiation and proliferation of lymphocytes as well as the development of immunoglobulin and enhances the ability of the human body to produce antibodies such as IgM and IgG. Immunoglobulin and antibody synthesis are hindered by a lack of Se (Xia et al., 2021[73]). Broilers that received 1.50 mg/kg of dietary SeNPs had greater IgG and IgA titers during both the secondary and primary immunological responses against blood cells one day after hatching. ROS produced by neutrophils can be used to destroy bacteria. Leukotriene B4 production, which is essential for neutrophil chemotaxis, is impaired by Se deficiency but can be improved by Se supplementation. Nutritional Se intake has a direct and indirect impact on NKs activity (Tsuji et al., 2021[67]). The cytotoxic effect of NKs has been found in numerous investigations to be significantly influenced by dietary Se. A study of more than 300 North American men found that supplementation with Se boosted plasma Se levels, and there was a positive association between both the plasma concentration of Se and the proportion of NKs in the bloodstream. Serum Se levels are favorably associated with the number of CD16+ NKs in the blood plasma of aged adults (Xia et al., 2021[73]).

### Selenium for bone stability

The health of the skeletal system is crucial for the elderly. The ability to have a thorough grasp of the association between Se and bone strength is beneficial when developing early-life therapies (Zeng et al., 2013[84]). SelPs expressed in human embryonic osteoblasts would seem to protect the bone from OS, which might also contribute to the development of osteoporosis by suppressing osteoblastic proliferation of bone marrow stromal cells. Se, being a crucial ingredient of SelPs, is far more likely to play a critical role in the connections between Se and bone mineral density (BMD) (Beukhof et al., 2016[10]). To the best of our knowledge, there have been at least ten studies that have looked at the relationship between nutritional or serum Se concentrations and BMD, osteoporosis, or osteoporotic fractures. A lack of Se is related to loss of bone mass in male rats and osteoarthropathy in Kashin Beck disease (KBD) (Yang et al., 2022[77]). This is because Se shortage interferes with the manufacture of many antioxidant SelPs, which compromises bone metabolism and causes osteoarthropathy. Yao et al. (2011[79]) investigated the effects of supplemental Se mixed with iodine, which was developed for a regimen for the KBD endemic regions, on the histology of bones and development of plates cartilage in Wistar rats of both sexes. They suggested that the Se and iodine supplementation in rats resulted in the reduction of necrosis of the chondrocytes throughout their development and trabecular bone formation. Additionally, they noticed increases in the bone-to-tissue volume fraction, trabecular width, and trabecular number, as well as decreases in the trabecular gap between the bone and the tissue.

### Toxicity due to Selenium intake 

Continuous intake of high Se-containing foodstuffs or water can lead to Se accumulation and selenosis in the body, therefore, excessive Se intake is harmful to the body (Yang and Liu, 2017[76]). Se toxicosis may affect any kind of animal, according to experts. While this is the case, poisoning is more common in foods such as bovine species, sheep and horse species, as well as other plant herbivores that graze on plants with an excess amount of selenium (Loh et al., 2020[34]). Apart from that, since most plant species have low Se concentrations, save for those that accumulate Se and are not traditionally used as feedstuffs, or those that grow in seleniferous soil, the toxicity of grazing plants is less likely to occur (Bano et al., 2021[5]). The effects of acute Se poisoning might include brain problems, changes in mental state, gastrointestinal symptoms, breathing signs, hepatocellular necrosis, renal failure, heart attacks, and other cardiac diseases, among other symptoms. According to certain studies, the most severe cases of Se intoxication might cause animals to develop at a slower rate than usual (Yang and Liu, 2017[76]). A study on Se poisoning in domestic animals found that feeding naturally occurring Se-containing foods with 25-50 mg Se/kg increased conception and fetal resorption rates in cows, sheep, and horses. The dosages would have been around 0.5-1.5 mg Se/kg/day if big animals consume about 2 %-3 % of their body weight. Hair loss, lameness, degeneration of the heart, liver, and kidneys, and fibrosis were some of the additional side effects of such high Se levels (MacFarquhar et al., 2010[37]). It has been discovered that cystic ovaries are linked to blood Se concentrations of >108 ng/mL in 136 Holstein cows from four flocks. Milk from control cows had higher levels of progesterone than milk from animals given Se therapy, but no information was supplied on how much Se each of the cows received (Mohammed et al., 1991[44]). Estrus cycle duration and behavior, progesterone and estrogen profiles, and pregnancy rates were not affected by alfalfa granules containing Na_2_SeO_4_ (24 ppm) or *Astragalus bisulcatus* (29 ppm) as an Se input for 88 days, from 52 days before pregnancy to day 28 of pregnancy. Amounts of food consumed were not recorded, and the report indicates that the food supply was restricted to ensure that it corresponded to that consumed by those in the group with the lowest intake level. The Water Buffalo of the Indian Punjab report similar symptoms because of high Se levels in soil waters (Loomba et al., 2020[35]). If Se-rich soils are used to produce pigs, fish, and other grain-eating animals, then poisoning may also occur owing to feed formulation mistakes. It is also well known that excessive consumption of Se by females during egg production might have detrimental effects on embryonic fish and birds. Se consumption in chickens and fishes may cause mutations in these embryos, making them particularly vulnerable to this kind of mutation (Nasr-Eldahan et al., 2021[46]). Moreover, Se toxicosis is rare in small animal pets but can happen upon ingestion of Se possessing skincare products or Se supplement tablets. Se toxicity may be affected by a wide range of factors, but in general, an oral acute Se dosage of 1-10 mg/kg/Bw (Body weight) is deadly for the majority of animals. Puppies, calves, lambs, and dogs may all die at dosages of as little as 1 mg/kg/Bw of parenteral Se preparations, which is why these products should never be given to young animals. Younger animals are more vulnerable to Se poisoning, and the chemical forms may have different toxicity depending on the age of the animal (Yang and Jia, 2014[75]).

## Conclusion

There have been significant advances in the understanding of the import and control of the trace element Se in cell biology, biochemistry, and molecular biology in recent years. Se toxicity, with a narrow therapeutic window, makes it necessary to avoid overconsumption of Se supplements. New research shows the need to maintain an optimal Se status for health. As far as molecular aspects are concerned, we are eager to learn more about Se-dependent chemoprevention. Research into the effects of Se and SelPs will aid in the development of novel medicinal approaches. Specifically, ebselen, an organo-Se compound that mimics GPX, has been shown to suppress superoxide anion formation and release of NO as well as to scavenge peroxynitrite and protect against lipid peroxidation, which is consistent with its proposed ability to prevent OS. Metabolic processes relating to SeMet and Se remain mostly unknown. SeCys insertion into protein is a complex process, and while many of the variables involved have been identified, it is still not clear how it all works. As a result, despite substantial attempts to investigate the positives and negatives of Se in clinical testing, key impediments remain such as significant gaps in our understanding of the metabolic actions of Se and SelPs. Greater knowledge of these fundamental processes will aid in the design and evaluation of safe and successful human trials and contribute to novel treatment interventions.

## Declaration

### Acknowledgment

This study was co-financed by the Preludium Bis 2 (2020/39/O/NZ9/00639) from the National Science Centre (NCN), Poland.

### Conflict of interest statement

The authors declare no conflict of interest.

## Figures and Tables

**Table 1:**
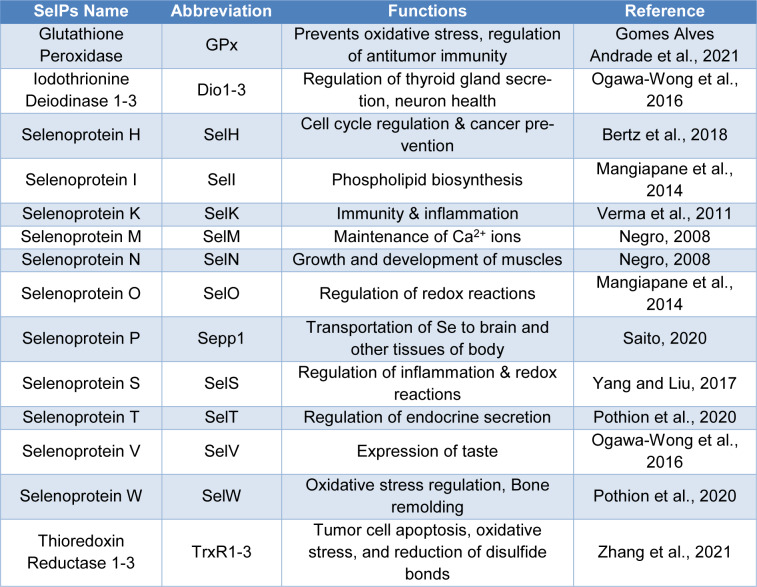
Mammalian Selenoproteins (SelPs) and their functions in the body
